# Models for the Design and Optimization of the Multi-Stage Wiredrawing Process of ZnAl15% Wires for Spray Metallization

**DOI:** 10.3390/ma17215307

**Published:** 2024-10-31

**Authors:** Juan Carlos del Rey, Guillermo Guerrero-Vacas, Francisco Comino, Oscar Rodríguez-Alabanda

**Affiliations:** Department of Mechanics, Higher Polytechnic School, University of Córdoba, Leonardo da Vinci Building, Rabanales University Campus, Madrid-Cadiz Road, km 396, 14014 Córdoba, Spain; z32remej@uco.es (J.C.d.R.); francisco.comino@uco.es (F.C.); orodriguez@uco.es (O.R.-A.)

**Keywords:** wiredrawing ZnAl15%, FEM, optimal die angle, formability, friction coefficient

## Abstract

Metallization, a process for applying anti-corrosion coatings, has advantages over hot-dip galvanizing, such as reduced thermal stress and the ability to work “in situ”. This process consists of the projection of a protective metal as coating from a wire as application material, and this wire is obtained by multi-stage wiredrawing. For the metallization process, a zinc–aluminum alloy wire obtained by this process is used. This industrial process requires multiple stages/dies of diameter reduction, and determining the optimal sequence is complex. Thus, this work focuses on developing models with the aim of designing and optimizing the wiredrawing process of zinc–aluminum (ZnAl) alloys, specifically ZnAl15%, used for anti-corrosion applications. Both analytical models and numerical models based on the finite element method (FEM) and implemented by computer-aided engineering (CAE) software Deform 2D/3D v.12, enabled the prediction of the drawing stress and drawing force in each drawing stage, producing values consistent with experimental measurements. Key findings include the modeling of the material behavior when ZnAl15% wires were subjected to the tensile test at different speeds, with strain rate sensitivity coefficient *m* = 0.0128, demonstrating that this type of alloy is especially sensitive to the strain rate. In addition, the optimal friction coefficient (µ) for the drawing process of this material was experimentally identified as µ = 0.28, the ideal drawing die angle was determined to be 2α = 10°, and the alloy’s deformability limit has been established by a reduction ratio r ≤ 22.5%, which indicates good plastic deformation capacity. The experimental results confirmed that the development of the proposed models can be feasible to facilitate the design and optimization of industrial processes, improving the efficiency and quality of ZnAl15% alloy wire production.

## 1. Introduction

When zinc is applied as a coating, it creates a physical barrier that prevents corrosive elements from reaching the underlying metal, and this coating can be applied through various processes such as hot-dip galvanizing, electroplating, or thermal spraying.

Hot-dip galvanizing or batch galvanizing consists of dipping steel components into molten zinc, forming a thick and durable layer. This method is widely used in the construction industry for protecting structural steel, such as beams, columns, and rebar, as well as in the production of outdoor structures like fences, guardrails, and utility poles, but it is a process that is carried out in expensive industrial facilities where space is limited [1]. Moreover, in the electrogalvanizing processes, zinc is deposited onto steel using an electrical current. The resulting coating is very thin but is still effective for corrosion protection in applications such as the coating of automotive body panels, household appliances, or electronic components.

Thermal spraying involves spraying molten zinc particles onto a surface using either flame metallization or electric arc technologies. This process is versatile, allowing its application “in situ” and on large or complex structures, without limits of space or shapes. It also enables easy repairs and touch-ups on welds, damaged areas, or cut edges. The resulting coatings are thick, durable, and highly resistant to mechanical damage, with the added benefit of not causing thermal deformation of the coated parts.

The addition of aluminum to zinc wire in cold spraying processes results in a coating that not only retains the protective qualities of zinc but also benefits from aluminum’s superior corrosion resistance, mechanical strength, thermal stability, and cost-effectiveness [2]. Zinc–aluminum metallizing coating combines the advantages of the two metals but also gives a more economical solution: for the same surface to be treated and coat thickness, about 30% less wire is required, and it is safer to use since the residue volume generated in the metallizing process is lower with fewer dusts in the spray booth [3]. This combination makes zinc–aluminum coatings an attractive choice for a wide range of industrial applications where long-lasting protection and performance are required, particularly in marine or industrial environments where exposure to corrosive elements like saltwater or chemicals is high [4,5]. Furthermore, aluminum in the alloy can improve the adhesion of the coating to the substrate, reduces the overall density/weight, and improves thermal stability and cathodic protection, allowing better surface finishing with cost savings compared to pure zinc.

The wire used in thermal spraying is supplied in the form of coils/drums, and a poor quality ZnAl wire can lead to a range of problems, from inconsistent coating performance to coating equipment malfunction because of an inconsistent diameter or poor surface quality of the wire that can cause locks or breakages. Then, the optimum conditions during the cold forming process by wiredrawing is the key to achieve a high-quality raw material for its use in the thermal spraying process.

The plastic deformation condition of zinc alloys is quite particular since zinc could facilitate a plastic–superplastic behavior, which is translated to a dynamic restoration/recrystallization of its structure at significantly lower temperatures than those in the case of other metals that require high temperatures to be deformed, around 150 to 300 °C (423 to 573 K) [6,7]. Then, these conditions can especially occur in processes involving a high deformation rate, such as cold forming by wiredrawing, in which the strain rate may be as high as 10^4^ s^−1^. Furthermore, consulted documents corroborate that zinc alloys could easily reach adequate temperature conditions when cold worked at high deformation rates, since they allow the local temperature in the forming zone to increase [6,8] and then, when a high strain rate is applied, zinc wire can be adiabatically heated up to temperatures of around 120 °C and its behavior becomes superplastic [9,10,11]. This is not the case when Zn and ZnAl alloys are subjected to a low strain rate cold forming process, such as that produced by a conventional tensile test at room temperature. Thus, the present work, unlike other previous works discussing Zn and ZnAl alloy wiredrawing [12,13,14], has demonstrated how the strain rate affects the drawing force (F_d_) and required power (P) in the wiredrawing process when working at medium-low drawing speeds.

Analytical and numerical methods have been postulated as very useful tools to predict output variables, such as drawing stress and drawing force in the wiredrawing process, even reporting valuable information about stress and strain distributions in the forming zone during the process as well as residual strain distribution and damage in the drawn product or temperature distribution both in the wire and in the drawing die, alongside other possibilities of analysis. Nevertheless, all these models must be supported by rigorous experimental results to establish both the wire material behavior and the die wire–die system with all the boundary conditions that affects those output responses.

Analytical models of the wiredrawing process are mainly based on the slab classical method developed by Sachs in 1927 [15], which is based on the definition of an equilibrium of forces applied to a thin section of the wire (slab) assuming that the deformation field is uniform across the height of the slab and the normal stress is uniformly distributed on the plane parallel to it, defining the drawing stress during the process (σ_d_) and, in the end, the drawing force. Later, other authors like Wistreich [16,17] and Wright [18] determined the influence of the die geometry on the inhomogeneous deformation of the drawn wire, introducing the effect of a redundant work factor (Φ) in the determination of the drawing stress. Thus, the value of the redundant work factor was obtained with a semi-empirical approach and depends on the deformation zone shape (Δ). These analytical models involve the uniform work required, non-uniform redundant work, because of the material distortion and frictional work according to the Coulomb sliding model. However, the additional friction work due to the presence of the bearing length below the reduction cone at the exit of the die must be considered. Previous research has demonstrated the reliability of these analytical models for predicting the drawing force (F_d_) in wiredrawing but, while these models have been effectively improved and optimized, experimental comparisons indicate that the analytically determined values of F_d_ tend to be always lower than the experimentally measured values [19,20].

On the other hand, current numerical simulation software tools not only allow a more precise determination of the drawing force (F_d_), but also allows one to analyze the effect of geometric and technological variables in a much more detailed manner. In this line, a recent work conducted by di Donato et al. [21] developed numerical models based on finite element simulation (FEM) by a commercial code software demonstrating that the model allows one to predict the stress distribution, drawing stress, and strain distribution on the wire during the wiredrawing process of the copper wire with agreement with experimental data. The wiredrawing of steel alloys also has been studied previously by FEM simulation models in the past. Radionova et al. [22] analyzed stress and strain distributions in a multi-stage wiredrawing process of a grade 10 steel, demonstrating how the die angle directly affects these outputs since the die geometry conditions the tractional/compressive character of the forming process. Aluminum alloys have also been studied using FEM modeling and simulation techniques. A recent work of Kajino et al. [20] demonstrated that in the wiredrawing process of AA1070 and AA8079 aluminum wires with both angular and radial-shaped drawing dies, the redundant work effect that is associated with the die geometry has a strong effect on the drawing force at higher die semi-angles (α > 7°), while frictional work (contact, lubrication) has a strong influence when die semi-angles are lower (α < 7°). Another recent work made by Milenin et al. [23] analyzed the influence of the die semi-angle on the surface quality of the pure zinc wire by FEM simulations, improving the surface quality when working with an atypical low die semi-angle of α = 5° and showing the relationship of this quality with a lower value of the strain near the wire surface. However, the wiredrawing processes of zinc and zinc–aluminum alloys have been scarcely studied using this methodology, and no other studies have been found that model the behavior of zinc–aluminum alloys during wiredrawing.

There are two fundamental keys to achieve optimum results in the development of simulation models of the wiredrawing process: (i) the strain hardening model/law of the drawn material and (ii) the friction conditions at the wire–die contact interface. Then, it is crucial to experimentally determine the strain hardening model that defines the behavior of the metallic material when it is processed by cold forming. Previous works demonstrated different behavior models depending on the elasto-plasticity of the material. A linear behavior model could be implemented in the case of alloys with a high strain hardening such as low carbon steels [24] while exponential behavior model is common in the case of plastic metals like copper [25]. As previously stated, considering the special behavior of zinc as a metal that is very sensitive to the rate of deformation, ZnAl alloys require a special consideration in this. Thus, a strain hardening behavior model that considers the strain rate ($\dot{\varepsilon }$) applied during the forming process must be implemented since an increased strain rate normally causes a higher flow stress. On the other hand, it is possible to consider that, under ideal lubrication and speed conditions, the contact between tungsten carbide (drawing die) and the Zn or ZnAl wire can be reduced to values around µ = 0.1 ÷ 0.2 [26,27]. Aristides Santana-Martínez et al. [27] have determined the variability of the value of µ by means of experimentally measuring the drawing force in the wiredrawing process of electrolytic copper and applying Avitzur’s relationship, which shows the dependence of μ on the geometry of the die and the yield stress of the alloy (σ_Y_) and the drawing stress (σ_d_). However, the contact conditions between the wire and die in a wiredrawing process are quite particular, so it is very important to determine the precise value of the friction coefficient at this wire–die interface by inverse experimentation.

The main objective of this study was to develop a comprehensive simulation model for the multi-stage wiredrawing process of ZnAl15% alloy wire, addressing the specific challenges posed by its sensitivity to strain rate and deformation speed. Unlike previous works, this study not only defines the strain-hardening behavior of the ZnAl15% alloy but also integrates experimental data to optimize friction conditions at the wire–die interface. As a result, the proposed model offers a novel and improved framework for analyzing, designing, and optimizing the sequential wiredrawing processes of ZnAl15%, contributing valuable insights into the process limits and opportunities for efficiency gains.

## 2. Materials and Methods

The material to be modeled is a 2.50 mm diameter wire of ZnAl15% alloy supplied by Zinacor company (Zinacor, S.A., Angleur, Belgium). This raw material wire has been manufactured from virgin special high-grade zinc and aluminum ingots through a Properzi continuous casting [28,29] process that ensures compliance with the specifications required by the standard UNE-EN ISO 14919:2024, which specifies the purity and quality of wires for the manufacture of wire for thermal spraying [30]. The Properzi process begins with molten zinc and aluminum in a shaft furnace, followed by casting onto a copper wheel. The resulting bar, with a square cross-section of 460 mm^2^, is then passed through a rolling mill consisting of nine stands, each with three rolls arranged at 120-degree intervals. This process reduces the bar to a final rod with a diameter ranging from 4.25 to 5.58 mm. The rod is subsequently processed by wiredrawing and packaged into coils weighing 15–30 kg or drums weighing 200–250 kg. According to the supplier’s quality certificate [31], ZnAl15% alloy has a density of 5.73 g/cm^3^, a melting point of T_M_ ≈ 382–450 °C, with an ultimate tensile strength ranging from 110 to 150 MPa and a percentage of elongation after rupture ranging from 70% to 200%. The chemical composition of the alloy is shown in Table 1.

**Figure 1:**
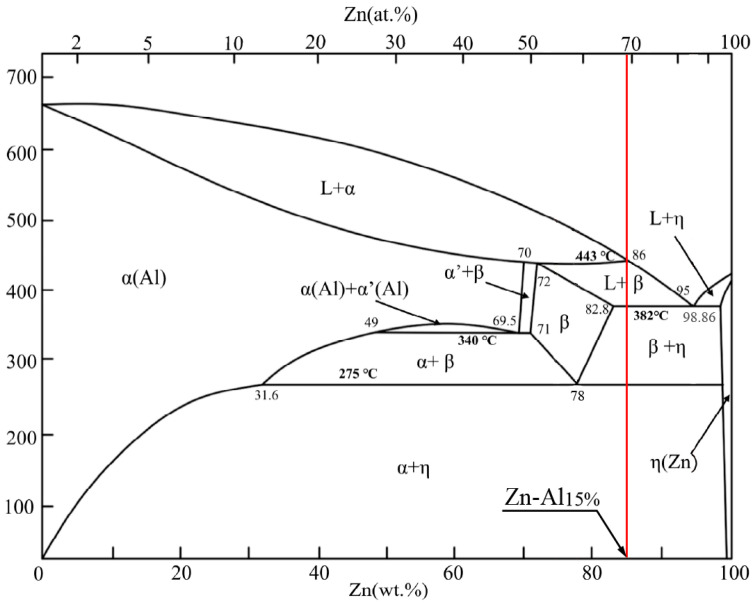
Phase diagram of the Zn–Al alloy and Zn-Al 15% alloy [32].

Figure 1 shows the phase diagram for the Zn–Al alloy. As can be observed in the consulted work of Zhai et al. [32], during the cooling process of the ZnAl15% alloy, the following reactions occur: (i) the transition between liquid + pro-eutectic solid (α) and liquid + peritectic solid (β) begins at 443 °C, and below this temperature at the limit of 382 °C the alloy is transformed from liquid + peritectic solid (β) to a bi-phase eutectic solid (β + η). According to the phase diagram, the ZnAl15% alloy rod obtained by continuous casting exhibits the structure bi-phase eutectic solid (β + η). It must be noted that, during the cold wiredrawing process, the alloy wire is under 275 °C and, therefore, no phase transformation that could alter the mechanical properties or microstructure of the alloy is expected.

### 2.1. Mechanical Properties and Strain Hardening Behavior

The behavior regarding hardening by cold plastic deformation of Zn and ZnAl alloys is dependent on the strain, strain rate, temperature, and the grain size/orientation of the raw material. Also, the material flow stress usually increases with increasing strain rate and decreases with a higher temperature. Thus, the governing law of the material behavior has been defined by Equation (1), which considers a strength coefficient (K), the value of the average true strain rate applied in the forming process $\left(\dot{\varepsilon }\right)$ and the strain rate sensitivity coefficient (*m*), the sensitivity that the alloy shows when it is deformed at different speeds [8,33]. For most metals at room temperature, the magnitude of *m* is quite low (between 0 and 0.03). The average true strain rate $\left(\dot{\varepsilon }\right)$ in a single wiredrawing pass could be determined by Equation (2), where d_0_ is the input diameter of the wire, d_f_ is the output diameter, A_0_ is the section area of the input wire, A_f_ is the output area, α is the die semi-angle, and v_0_ is the input speed.

(1) $$\sigma_{Y}=K\cdot {\left(\dot{\varepsilon }\right)}^{m}$$

(2) $$\dot{\varepsilon }=6\cdot \left[\frac{v_{0}\cdot d_{0}^{2}}{\left(d_{0}^{3}-d_{f}^{3}\right)}\right]\cdot \tan\alpha \cdot ln\frac{A_{0}}{A_{f}}$$

**Figure 2:**
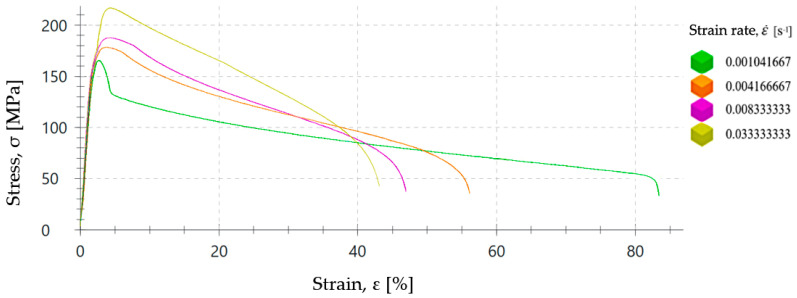
Stress–strain curves of ZnAl15% alloy wire of 2.50 mm diameter obtained from tensile tests performed at different testing speeds between 6.25 mm/min and 200 mm/min.

To assess the influence of the strain rate on the plastic forming process of the ZnAl15% alloy, a series of tensile tests have been carried out at room temperature and different speeds in a range between 6.25 and 200 mm/min to determine the influence of the strain rate on the value of the yield strength (σ_Y_). Thus, seven tensile tests with ZnAl15% wire specimens of 2.50 mm diameter with a gauge length of L_0_ = 100 mm were performed according to the ISO 6892-1:2020 [34]. The stress–strain curves are shown in Figure 2 and yield stress values measured in these tests can be seen in Table 2.

The values obtained for the yield stress limit (σ_Y_) are consistent with those determined in previous consulted works in which this alloy has been studied [10,14]. Also, it has been found that by using low aluminum additions to the zinc wire, its yield limit and tensile strength can be increased [12,23]. Specifically, from these works it could be stated that the yield stress limit of pure zinc wire could be increased from σ_Y(pure Zn)_ ≈ 60 MPa to σ_Y(ZnAl15%)_ ≈ 110 ÷ 150 MPa when 15% of aluminum is added together with zinc in the form of an alloy.

On the other hand, Equation (3) expresses the relationship to convert testing speed (v) from mm/min to strain rate ($\dot{\varepsilon }$) in s^−1^, where L_0_ is the gauge length.

(3) $$\dot{\varepsilon }\left[s^{-1}\right]=\frac{v\left[\frac{mm}{min}\right]\cdot \frac{1}{60}\left[\frac{min}{s}\right]}{L_{0}\left[mm\right]}$$

**Figure 3:**
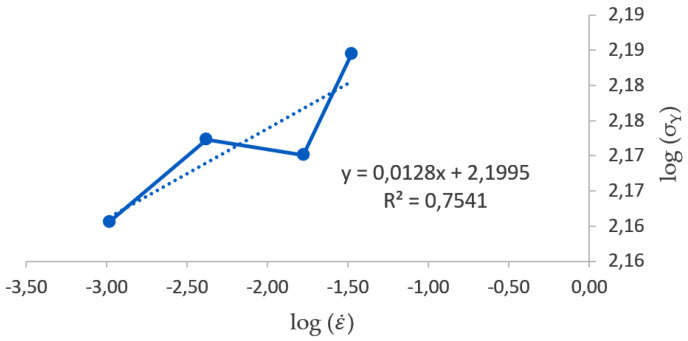
Plot of the log–log graph for yield limit (σ_Y_) vs. strain rate ($\dot{\varepsilon }$).

Moreover, the log–log graphic representation of strain rate ($\dot{\varepsilon }$) vs. yield limit (σ_Y_) shown in Figure 3 represents a linear function used to determine the value of the strain rate sensitivity coefficient (*m*) by the least squares method. It must be noted that *m* is the slope of the trend line, and, for a coherent calculation of the strain rate sensitivity coefficient (*m*), it must be positive. Thus, the yield stress data corresponding to speed rates at 6.25, 5, 50 and 200 mm/min have been allowed in order to determine this trend.

Once the value of the strain rate sensitivity coefficient has been determined as *m* = 0.0128, the strength coefficient could be calculated by Equation (1), obtaining a value of K = 158.30 MPa. Then, the behavior of the ZnAl15% wire could be expressed as a general relationship (Equation (4)), where it is possible to determine the value of the flow stress (σ_Y_) as a function of the strain rate, $\dot{\varepsilon }$, at room temperature.

(4) $$\sigma_{Y}=158.3\cdot {\left(\dot{\varepsilon }\right)}^{0.0128}$$

It must be noted that no previous works have been found about the experimental definition of the strain rate sensitivity coefficient (*m*) and strength coefficient (K) of ZnAl15% wires considering the influence of deformation speed. In this sense, Quintana et al. [35] determined a value of the strain rate sensitivity coefficient (*m*) for Cu–Ti alloyed zinc, such as *m* = 0.077 ÷ 0.080, since Cu and Ti were added to make the alloy harder for rolling purposes, which demonstrates the coherency of the value of *m* when zinc is alloyed with aluminum, since Al is a softer alloying element compared to Cu–Ti. Nevertheless, higher *m*-values above 0.1 are commonly associated with certain temperature conditions in the plastic forming process (warm to hot forming), promoting larger elongations by preventing localization of the deformation as a sharp neck that occurs generally in a range of temperatures between 30% and 75% of the melting temperature of the material (T_m_), while lower m-values are associated with most metals and alloys at room temperature, including zinc and zinc alloys [11].

### 2.2. Wiredrawing Sequence Object of Study

The multi-stage wiredrawing sequence under study was proposed by the machine manufacturer, Victory Technology International Ltd. (Victory Technology International Ltd., Dongguan, China) [36], which provided the technical data detailing the technological setting recommended for ZnAl wire processing. The industrial process setup is shown in Table 3. It is important to note that the wiredrawing process is classified as a manufacturing method involving a combination of tensile and compressive conditions. Specifically, it is defined as a drawing process through restricted tool orifices, as outlined in the DIN 8584-2:2003-09 standard [37].

This wiredrawing sequence has been taken as a starting point for the implementation and fine-tuning of the analysis models developed in this work.

### 2.3. Analytical Determination of the Drawing Force

The slab method involves analytical modeling of the plastic deformation zone within the drawing die, accounting for homogeneous deformation, frictional work, and additional work required to consider the effects of inhomogeneous deformation due to material distortion, as the material layers near the contact surface require extra work to change the flow direction when the wire enters the reduction cone [17,18,21]. Additionally, the cylindrical bearing section (L_c_) located just after the reduction cone is designed to calibrate the drawn product’s cross-section and to improve wire surface quality, also affecting the drawing stress because of friction effect [38]. Considering all these factors, Equation (5) has been proposed as the most complete analytical model to calculate the drawing stress (σ_d_) in each stage/drawing pass (i) of a sequential multi-stage wiredrawing process.

(5) $$\sigma_{d_{i}}={\bar{\sigma }}_{Y_{i}}\cdot \frac{\left(1+B\right)}{B}\left[1-{\left(\frac{d_{fi}}{d_{0i}}\right)}^{2B}\right]\cdot \Phi +\mu \cdot \frac{{2\cdot L}_{ci}}{d_{fi}}\cdot {\bar{\sigma }}_{Y_{i}}$$

On the other hand, the effective plastic deformation zone of a conical die shape, which depends on area reduction (r) and die semi-angle (α), is characterized by a shape factor (∆) that is defined in Equation (6). The reduction ratio is expressed by Equation (7) and Equation (5) corresponds to the effect of redundant work dependent on the additional inhomogeneous deformation factor (Φ) that is defined by Equation (8) for typical drawing passes (∆ ≥ 1) or with higher die angles and/or lower drawing reductions [17,18].

(6) $$∆=\frac{d_{m}}{L}=\frac{d_{0}+d_{f}}{d_{0}-d_{f}}\cdot sin\;\alpha =\frac{\alpha }{r}\cdot {\left[1+\sqrt{1-r}\right]}^{2}$$

(7) $$r=1-{\left(\frac{d_{f}}{d_{0}}\right)}^{2}$$

(8) Φ=0.8+∆4.4

### 2.4. Lubrication and Friction Coefficient

Water-based synthetic or semi-synthetic lubricants are commonly used in the wiredrawing processes of non-ferreous alloys due to their beneficial properties in terms of lubrication, cooling, and chemical stability. In this work, Bestril Al-200 (Brugarolas, Barcelona, Spain) synthetic oil, which is a medium viscosity oil (210 mm^2^/s at 40 °C) indicated for thin and medium wiredrawing with non-immersed dies, has been applied directly in the entrance cone into the die during each of the wiredrawing tests of short segments of ZnAl15% alloy specimens.

In the wiredrawing of ductile metals and alloys, the coefficient of friction (μ) can vary significantly based on factors such as the lubrication quality, material properties, and drawing conditions. Then, when the deformation zone is adequately lubricated, the coefficient of friction could be typically reduced within the range of μ = 0.1 to 0.3 between a ductile alloy and tungsten carbide [26,27,39]. Thus, for initial modeling purposes, the die–wire sliding friction coefficient has been stablished as μ = 0.1, assuming the best boundary condition of the lubricant film into the reduction cone during the process.

In practice, measuring the friction stress and determining the coefficient of friction or friction factor acting at the wire–die interface is a challenge. Wistreich et al. [16] have used a split die technique, where through measuring the force with which the die parts are separated and the stretching force, it becomes possible to directly determine the friction shear stress. Then, a simpler approach to calculate the coefficient of friction is by clearing this constant in the drawing tension calculation Equation (9), as proposed by Wright [18].

(9) $$\sigma_{d}=\sigma_{Y}\cdot \left[\left(3.2/∆\right)+0.9\right]\cdot \left(\alpha +\mu \right)$$

In the present work, the value of the yield stress limit of the material (σ_Y_), the stretching stress and the shape factor (σ_d_) have been experimentally determined, which has allowed empirical determination, by means of the equation proposed by Wright, the value of the friction coefficient (µ) that acts at the die–wire interface during the tests.

### 2.5. Implementation of the Finite Element Simulation Model

The Deform 2D/3D v.12 software application (SFTC, Columbus, OH, USA) was used to implement the finite element method (FEM) for modeling and simulating each pass of the wiredrawing process [40]. Thus, the drawing force (F_d_) has been determined in each drawing stage as a function of the drawing stress (σ_d_) observed and the output section of the drawn wire in the simulations.

The strain hardening behavior of the drawn ZnAl15% alloy wire has been implemented by means of Expression (4), ensuring consideration of the effect of strain rate on the condition of the processed material.

For modeling, the drawing die was defined as a perfectly rigid body, while the wire was defined as a perfectly plastic body, both in an axisymmetric system, with an initial mesh consisting of approximately 3000 elements over a 20 mm segment. The displacement of all nodes located on the wire’s symmetry axis was restricted in the drawing direction and automatic remeshing scheme was active throughout the resolution of the numerical problem. Furthermore, the wire’s movement was modeled as being pulled along the drawing direction, setting a constant drawing speed (v) to all nodes at the end of the wire at the exit of the die. The Coulomb sliding friction (µ) was stablished between the wire and the die.

The FEM simulation allowed for analysis of the whole multi-stage sequence. Thus, the material corresponding to the result of accumulated deformation has been considered as the starting wire at the beginning and for the entry into each of the dies in the modeling of each of the successive wiredrawing passes that constitute the multi-stage process. In this way, it has been possible to numerically simulate the accumulated deformation in the drawn product at the end of the process.

The developed FEM model has also allowed a numerical analysis to determine the optimal conditions in terms of the geometry of the dies (die semi-angle, α) and the limit value of section reduction ratio per pass (r) that can be applied in the sequential process.

### 2.6. Experimental Procedure

In the experiments, short ZnAl15% alloy segments of 150 mm length have been used, reducing their section by wiredrawing from a diameter of 2.50 mm to a diameter of 2.00 mm, applying a sequence constituted in 5 stages/dies that corresponds to the last five passes in the multi-stage process object of this study (Table 3).

Initially, 25 specimens of the raw wire of ZnAl15% with 2.50 mm in diameter were cut with a length of 150 mm and then pointed at one of their ends. In this way, each of the reductions to be applied implementing the last five stages/passes of the sequence under study have been repeated 5 times to determine the average values of the drawing force (F_d_): (i) the reduction with die nr. 13 (2.50 mm to 2.37 mm) was applied to the 25 specimens, (ii) the reduction with die nr. 14 (2.37 mm to 2.27 mm) to 20 specimens, (iii) the reduction with die nr. 15 (2.27 mm to 2.18 mm) to 15 specimens, (iv) the reduction with die nr.16 to 10 specimens, and (v) the reduction with die nr. 17 (2.09 mm to 2.00 mm) to the last 5 specimens.

**Figure 4:**
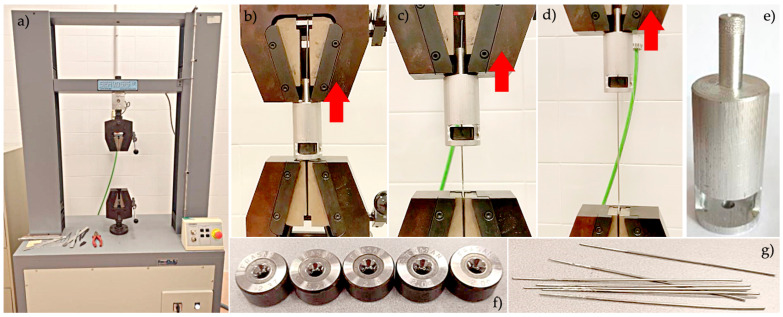
Experimental wiredrawing setup: (**a**) tensile press; (**b**–**d**) drawing process of the specimen; (**e**) die holder; (**f**) drawing dies; (**g**) ZnAl15% alloy wire specimens with 150 mm length and pointed. The wire is fixed in the lower claw and the red arrow indicates the upward movement of the die by the action of the upper traction claw.

Wiredrawing tests of these wire segments were performed on a tensile testing machine, the Servosis model ME-405 (Servosis, S. L., Pinto, Madrid, Spain), at a drawing speed of 3 mm per second (180 mm/min). Force vs. displacement data were registered by the control software PDC2k v. 2.0.0.800 application (Servosis, S. L., Pinto, Madrid, Spain). In each experiment, the wire specimen was passed through the die until the pointed part extended beyond it and then the drawing die was inserted into the die holder. The entire assembly is mounted on the machine such that the upper clamping claw grips the die holder from its top pin and the lower clamping claw grips the pointed wire, pulling at the exit from the die. The test starts in this position, causing the die to move upwards, drawing the wire and registering force versus displacement data. Figure 4 shows the experimental setup and tooling.

During the tests, the drawing force value (F_d_) was recorded as a tensile force and, knowing the area of the wire at the exit of each die, the drawing stress (σ_d_) was determined. The experimental results have been contrasted with those obtained by the proposed models to validate them.

Finally, tensile strength tests were performed on the specimens obtained after applying each of these five reductions. These mechanical tests allowed for determination of the possible mechanical impact on the ZnAl15% alloy when it is processed by low-speed multi-stage wiredrawing.

### 2.7. Theoretical Evaluation of the Maximum Area Reduction, Optimum Die Geometry, and Friction Coefficient

The maximum reduction per pass that a ZnAl15% wire can undergo has been determined through FEM simulation. For this purpose, simulations were performed at various reduction rates in a single drawing pass (8%, 15%, 20%, 25%, 35%, and 43%) until the wire failed due to tensile stress. The corresponding percentage elongation for each reduction rate was also calculated. The theoretical percentage elongation (e) for a given section reduction during drawing is defined by Equation (10), while the relationship described by Equation (11) correlates strain/unit deformation (ε) to the elongation (e) of the drawn wire, and Equation (12) throws the area reduction % in any given drawing pass [18].

(10) $$e=\frac{L_{f}-L_{0}}{L_{0}}$$

(11) ε=Ln(1+e)

(12) $$r(\%)=\frac{e}{1+e}\cdot 100$$

**Figure 5:**
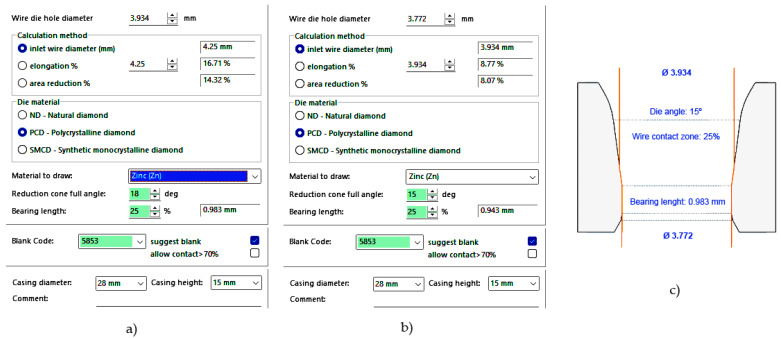
Information of the constructive parameters (elongation, reduction, bearing length, material, and die angle) of the drawing dies that the Drawing Die Wizard v. 2.02 software shows to process pure Zn wire: (**a**) from 4.25 mm to 3.93 mm (2α = 18°), (**b**) from 3.93 mm to 3.77 mm (2α = 15°), and (**c**) profile of the die showing the contact zone.

On the other hand, the Drawing Die Wizard v. 2.02 software (Figure 5) by the Esteves Group (Esteves Group Spain, Sant Just Desvern, Barcelona, Spain) [41] was used to determine the optimal geometric parameters for designing all the proposed drawing dies.

Using this tool, the values for the die semi-angle (α) and bearing length (Lc) were calculated, considering the input diameter (d_0_) and output diameter (d_f_) in each drawing pass of the wiredrawing sequence object of study. This software application has allowed us to determine an optimal value for the die angle 2α = 15°, which has been ratified by the supplier of the dies (Industrias Frasán, S. L., Miranda de Ebro, Spain) and supported by the previous works consulted [12]. In addition, a calibration length L_c_ = 0.25 · d_f_ was determined in all the drawing passes as indicated by both. All the geometrical data have been determined by this app and are exposed with the results in Table 1 and Table 2.

Additionally, the experimental friction coefficient (µ) could be determined by Equation (13) proposed by Wright [18,42] as a function of the experimental values obtained for the yield limit (σ_Y_) and drawing stress (σ_d_), the die angle (2α), and the shape factor (Δ) of the dies used.

(13) $$\mu =\left(\frac{\sigma_{d}}{\sigma_{y}}\right)\cdot \frac{1}{\sqrt{\frac{1}{3.2/∆}+0.9}}-\alpha$$

## 3. Results

In this section, results of drawing stress (σ_d_), drawing force (F_d_), and power (P) have been analyzed to understand the influence of geometrical parameters and drawing speed (v) on the process design and on the mechanical conditions of the drawn wire. For this, these technological parameters have been determined at low production rates of 0.003 m/s, comparing the results with those determined for a medium production rate of 2.667 m/s. Thus, the results obtained are based on the material behavior model defined as the sensitivity model ($\sigma_{y}=K\cdot {\dot{\varepsilon }}^{m}$) in which the coefficient of resistance of the material (K = 158 MPa) and a strain rate sensitivity exponent (*m* = 0.0128) are integrated. The values experimentally obtained for these coefficients agree with the findings of Quintana et al. [35].

In another way, the present study analyzed the optimum die angle (2α) and the maximum reduction ratio (r) theoretically admissible when working with a zinc–aluminum alloy to justify the selection of the geometrical characteristics of the drawing die, checking the reduction sequence applied in the multi-stage wiredrawing process object of this study. In addition, the results have been complemented with the determination of a working range for the friction coefficient (µ) in the wiredrawing process of the ZnAl15% alloy, based on the analytical, numerical, and experimental results obtained for the drawing stress (σ_d_).

Since the drawing process for pure zinc wire does not allow for an excessive reduction per pass, the industrial process object of the present study applied a low area reduction of about 8–9%. However, when alloyed with zinc, aluminum enhances mechanical strength and increases the alloy’s hardness. Thus, it is common to produce a ZnAl15% alloy wire under similar reduction conditions as those used for pure Zn wire.

### 3.1. Drawing Stress, Drawing Force and Required Power

The results obtained for the multi-stage wiredrawing process from the Ø4.25 mm wire rod to the Ø2.00 mm final wire at a low drawing speed (v = 0.003 m/s) are shown in Table 4, assuming µ = 0.1 as friction coefficient in ideal conditions. Furthermore, the power capacity of the industrial machine is 17 kW (allowing a maximum of 28 stages/dies), being 14.5 kW for a mechanical efficiency of 85% (courtesy of Victory Technology International Ltd., Dongguan, China [36]). However, P ≈ 0.2 kW has been estimated for the wiredrawing sequence at a low drawing speed, considering the geometrical conditions defined by a reduction ratio (r), die angle (2α), and bearing length (L_c_). The 1st to 12th stages have been analyzed both analytically and by numerical simulation (slab method (SLAB) and finite element method (FEM)), while the last five stages (13th to 17th) have also been defined experimentally.

**Figure 6:**
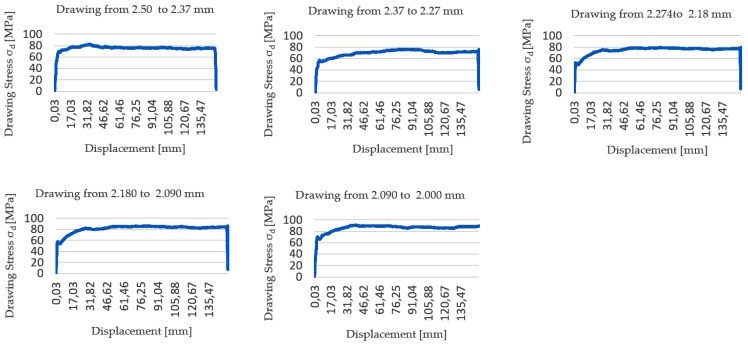
Experimental results of wiredrawing force during tests corresponding to the last five dies of the wiredrawing sequence: from 2.50 mm diameter to 2.00 mm diameter reduction.

Figure 6 shows the force vs. displacement graphs obtained during the wiredrawing experiments that have been performed in the tensile machine.

The results indicate that the models produce drawing stress values that align with those observed in the final five stages of the experimental wiredrawing sequence. This fact could be stated since both analytical and numerical models have given values slightly below those experimentally obtained.

**Figure 7:**
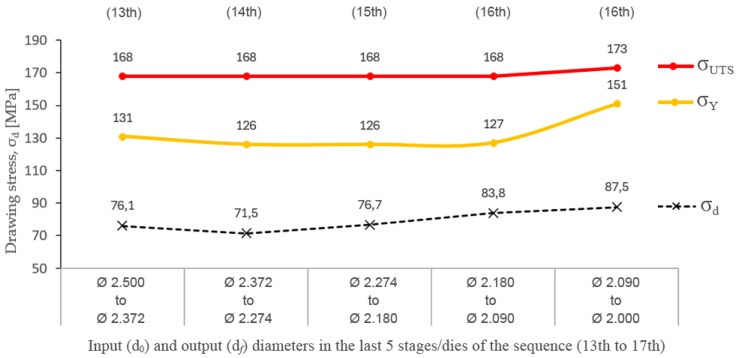
The experimental drawing stress (v = 0.003 m/s) vs. process limits established by the yield limit and the ultimate tensile stress in the last five reductions in the wiredrawing sequence object of study.

Figure 7 shows how the drawing stress values (σ_d_) experimentally measured are clearly lower than the process limits established by the yield limit (σ_d_ < σ_Y_ < σ_UTS_). As can be seen, they are still far from the mechanical limits of the alloy, which indicates that the wiredrawing sequence could be carried out in fewer drawing passes/stages without even exceeding this process limit, with the consequent saving of cost inherent in a lower number of drawing dies required.

It must be noted that in previous laboratory research that has been performed by Jabłoński et al. [12], pure zinc processed by discontinuous multi-stage wiredrawing reported drawing stress values (σ_d_) in the range between 30 and 90 MPa, close to the working range of the ZnAl15% alloy that is represented in the graph in Figure 6 but, in this case, pure Zn was processed in higher reduction ranges of 20% < r < 45% while, in the present work, it has been proven that ZnAl15% alloy only allows reductions below 22.5% to prevent wire necking at the exit of the die, as previously determined. This difference is because this pure metal has a significantly different mechanical behavior when subjected to tensile stress, showing a notably lower yield limit (σ_Y_) and ultimate tensile stress (σ_UTS_) than the alloyed ZnAl15%, but above all showing a much greater plastic period without necking than in the case of the alloy. Thus, the pure Zn behaves like a plastic material, showing a low value in the yield limit (σ_Y_) and a value of the ultimate tensile stress (σ_UTS_) when about 15–20% of tensile deformation is reached [12,43] while the addition of aluminum in the form of an alloy gives it an increase in its mechanical tensile strength at the expense of its plastic formability, as can be deduced by observing the proximity in terms of the percentage deformation point between the yield limit (σ_Y_) and the ultimate tensile stress (σ_UTS_) as it can be seen in the stress–strain curves obtained from the raw material (in Figure 1).

On the other hand, the developed models have been implemented to determine the drawing stress, drawing force, and required power when the production speed is set higher. Thus, Table 5 shows the results for the wiredrawing sequence considering a production speed of 2.667 m/s, values that have been analytically calculated and numerically simulated. In this case, P ≈ 10 kW has been estimated as the total power required to execute the sequential wiredrawing process.

As it can be observed, the results regarding the values of drawing stress (σ_d_) and drawing force (F_d_) that have been obtained by the analytical and numerical models, considering low production speed in all drawing passes (v = 0.003 m/s), increased only around 2–4% with respect to those obtained in the case of a process with a higher production speed of v = 2.667 m/s, although a notable increase was observed in the power requirements at higher drawing speed (v = 2.667 m/s). This means that, in the speed range studied, production speed has little impact on drawing stress and drawing force but significantly affects the power required in the process.

### 3.2. Die Geometry and Process Limits

Additionally, Deform 2D/3D v.12 FEM software has been used to simulate the final drawing pass (2.09 to 2.00 mm) at different angles, from 10° to 18°, to determine which is the angle (2α) that corresponds with the lower value of the drawing force (F_d_).

**Figure 8:**
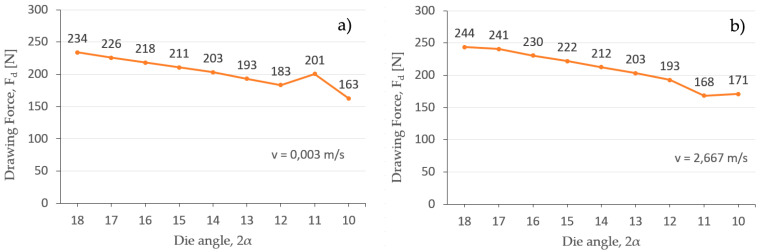
Drawing force (F_d_) at different die angles (2α), for a reduction from Ø 2.09 mm to 2 mm al low and high drawing speed: (**a**) 0.003 m/s and (**b**) 2.667 m/s.

Again, Figure 8 shows the results of the drawing force (F_d_) obtained for a low drawing speed v = 0.003 m/s and a high drawing speed v = 2.667 m/s, where the process speed (v) is not a factor that significantly affects the drawing force (F_d_) required in the process. Thus, the results shown in Table 4 and Table 5, obtained by both analytical and FEM simulation models, corroborate this fact. In this sense, Aristides Santana-Martinez et al. [27] obtained low variations of around 10% in the drawing force of fine copper wire when the drawing speed varies over a range of 1 to 3 m/s. Another work consulted [44] demonstrated that the drawing force in the multi-stage wiredrawing process of steel wire is also very insensitive to little variations in production speed between 1 and 5 m/s. Nevertheless, it must be considered that the cited works have shown higher influence of drawing speed (v) on the drawing force (F_d_) when working in industrial conditions that implicates drawing speeds even above 10 m/s.

In both cases, it can be seen that the die angle (2α) that allows the lowest drawing force value is 2α = 10°, considering a tolerance of +/−2° by the die supplier. To corroborate this fact, the value of Δ has been calculated by Equation (6) for all the combinations of possible reductions and die angles included in Table 6. Thus, the optimum shape factor (Δ_opt_) has been defined by Equation (14) proposed by Wright [18], considering the practical reduction of 8.43% that has been applied in the 17th stage of the wiredrawing sequence object of study. Then, the obtained value, Δ = 4.03, corresponds to a die semi-angle between α = 4° and α = 6°, confirming again that α ≅ 5° (2α = 10°) is the optimum die angle for an optimum geometric condition.

(14) $${∆}_{opt}=1.89\cdot \sqrt{\frac{\mu }{r}}{\left[1+\sqrt{1-r}\right]}^{2}$$

Since the objective is the design and optimization of the multi-stage wiredrawing sequence of the ZnAl15% wire, the reduction in stages (dies) in the sequence is conditioned by the maximum area reduction per stage/pass (r). To determine this limit, a series of simulations have been implemented establishing different values for the area reduction (r), considering as the ideal conditions: 2α = 10°, µ = 0.1, and drawing speed v = 2.667 m/s in all cases. Then, Table 7 shows the length increase measured from the simulations compared with the theoretical value. It was determined that the maximum allowable area reduction ratio is approximately r = 20–25%. Beyond this threshold, simulated elongation differs from the theoretical value by more than 5%, leading to an undesired reduction in the product’s exit diameter. In addition, the simulations provided evidence of undesired output and wire breakage when r > 40%.

**Figure 9:**
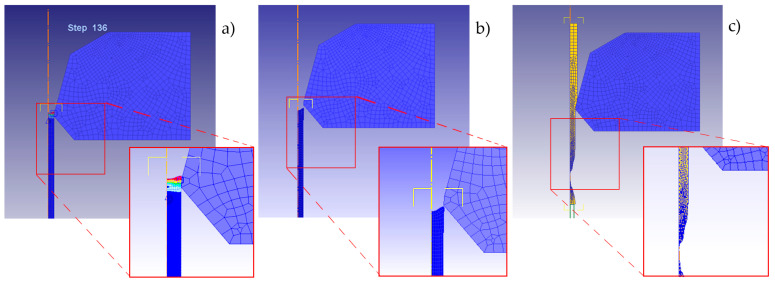
FEM simulation evidencing the wire elongation with an undesired output diameter in the cases (**a**) r = 20% (Ø_output_ ≈ 1.865 mm), (**b**) r = 35% (Ø_output_ ≈ 1.805 mm), and (**c**) the wire break when r = 43%, for a theoretical output to 1.894 mm diameter.

This fact is shown in the simulation’s graphics in Figure 9. Equations (10) and (11) allowed us to correlate the unit strain/deformation (ε) as a function of the elongation (e) of the drawn wire that has been measured in FEM simulations.

**Figure 10:**
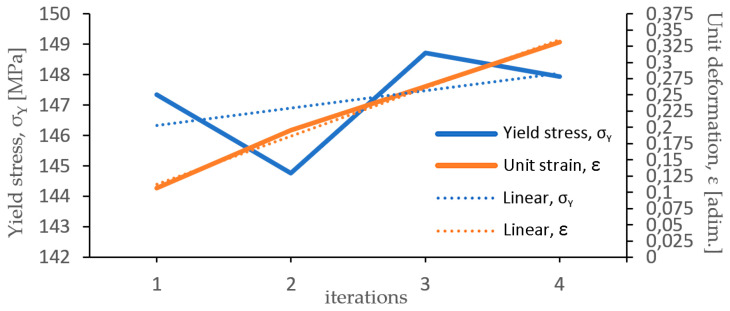
Yield stress (σ_Y_) values of a ZnAl15% alloy wire of 2.50 mm diameter vs. unitary deformation (ε) and the tendency lines of both variables.

Additionally, the double graph in Figure 10 shows the yield stress (σ_Y_) values obtained from the iterated tensile tests performed with 2.50 mm a ZnAl15% initial wire and the unit deformation (ε) corresponding to elongation (e) that has been measured from the FEM simulations. Furthermore, Equation (12) allows us to calculate the area reduction percentage as a function of elongation (e).

The point of intersection between tendency lines, corresponding to the evolution of yield value (σ_Y_) and unit deformation by wiredrawing (ε), gives rise to a value of ε ≅ 0.255, which determines the formability limit of the initial wire rod. Introducing this value in Equation (11), a value of e = 0.29 has been obtained. Then, Equation (12) allowed us to determine the value of maximum area reduction per pass r = 22.5%. This result, for a 2.50 mm diameter wire of ZnAl15% pre-processed by wiredrawing, is in concordance with the experimental findings of Knych et al. [14], which determined a limit value of the logarithmic unit deformation ε = 0.24 for a ZnAl15% wire rod that is 5.57 mm in diameter obtained by rolling from a Properzi-type continuous casting system [28,29], in which rolling is carried out in a 9-stands in-line rolling mill with a three-roller system at 120° (round–triangular–round), applying a lubricant/coolant emulsion during the process.

### 3.3. Estimation of the Friction Coefficient in the Wire-Die Interface

Since all the results, both analytical and by FEM simulations, have been obtained considering an ideal value of the friction coefficient µ = 0.1, it could be advised that the values of drawing stress (σ_d_) obtained by the proposed models are slightly lower that those experimentally obtained in the las five drawing passes/stages of the wiredrawing sequence object of this study that have been tested as reference. In this way, it can be assumed that real friction coefficient (µ) must be greater than µ = 0.1.

**Figure 11:**
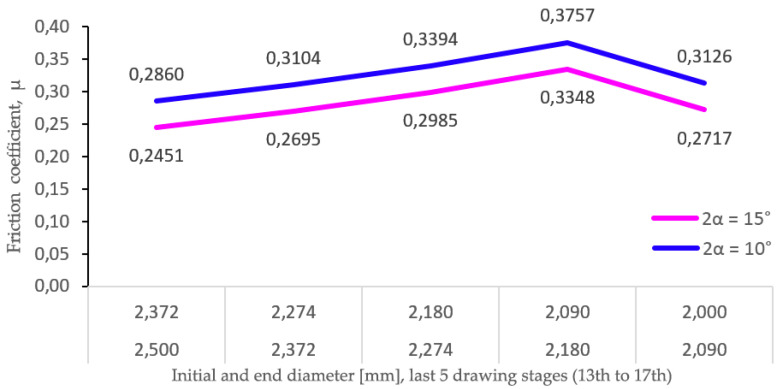
Evolution of the friction coefficient in two different scenarios (2α = 15° and 2α = 10°).

Using Equation (13) proposed by Wright, the friction coefficient has been determined, showing an average value of µ = 0.28, and the individual values for each of the last five drawing stages (2.50 to 2.00 mm) have encompassed values of 0.24, 0.27, 0.29, 0.33, and 0.27. Thus, Figure 11 shows the values of the friction coefficient (µ) obtained when Equation (8) is applied to two scenarios of die angle (2α = 15° and 2α = 10°), considering the drawing stresses and yield stresses obtained in the experimental reductions implemented in the last five wiredrawing stages of the sequence object of study (12^th^ to 17^th^ stages).

Based on the obtained results, it can be stated that the increase in the real friction coefficient (µ), which has occurred in the experimental tests carried out with the last five dies, is in line with the low-speed conditions (v = 0.003 m/s) and the low lubrication regime that occurs in such circumstances, in addition to the superplastic behavior of the processed alloy, which favors greater adhesion between wire and die metals.

**Figure 12:**
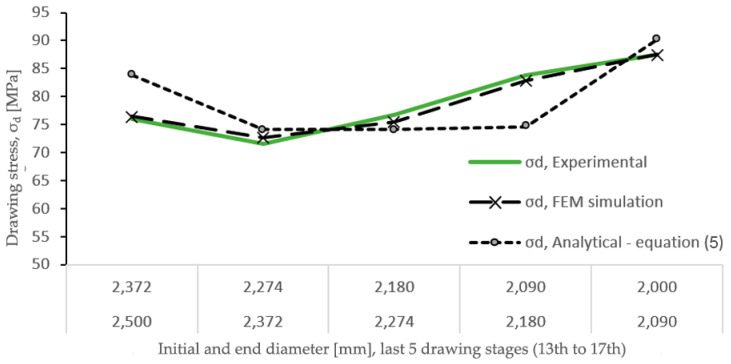
Drawing stress determined by analytical model and by FEM simulation (v = 0.003 mm/s, µ = 0.28 and die angle α = 15°) compared with experimental results.

Figure 12 shows the evolution of the drawing stress (σ_d_) in the last five reductions under study, determined analytically (Equation (5)) by FEM simulations and compared with those values obtained by experimental testing.

As can be seen by applying the previously determined real friction coefficient of µ = 0.28, the model based on numerical simulation yields drawing stress (σ_d_) values that are practically identical to those measured in the tests, while in the case of the calculation of the stresses using the analytical Equation (5), which has been based on the analytical slab method model, there are drawing stress (σ_d_) values that differ slightly from those measured experimentally. Specifically, in the 12th and 16th drawing passes, the values obtained analytically in the drawing stress have been 10.24% higher and 12.18% lower than those obtained experimentally, respectively, while in the case of those determined by the FEM simulation model, the difference with respect to the experimental values does not even reach 3% in any of the five stages analyzed. This fact shows that the model developed using finite elements is very reliable, while the analytical model allows attainment of drawing stress values with a margin of error of around ±10%.

In the industrial field, it is crucial to maintain low friction using advanced lubricants, coatings, and special dies. Nevertheless, there are certain products and processes where it is not always possible to keep friction minimal. In our case, low-speed tests were carried out by applying a synthetic oil as a lubricant at the entrance to the die, which, together with the superplastic properties of the alloy, further reinforces the hypothesis of a relatively high friction coefficient during the process.

### 3.4. Mechanical Analysis of the Drawn Material

**Figure 13:**
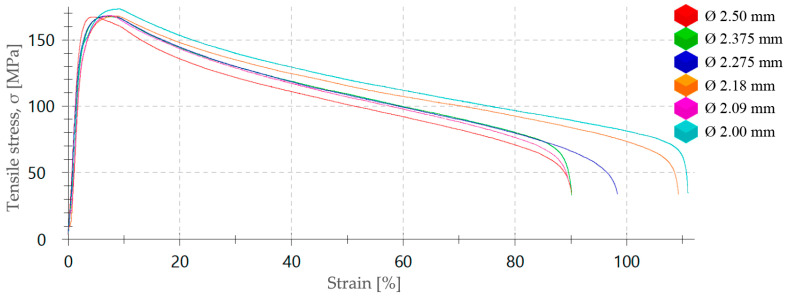
Stress–strain curves from tensile tests performed at 50 mm/min with ZnAl15% alloy wires corresponding to the last five stages from the wiredrawing sequence object of study.

Finally, it has been verified that, as with pure Zn, the ZnAl15% alloy is an alloy that, due to the high zinc content of its crystalline matrix, does not harden when deformed at room temperature, even at a low drawing speed (v = 0.003 m/s). This characteristic of zinc means that, during the wiredrawing process, which causes an increase in the temperature of wire, the ZnAl15% wire raises hot deformation conditions, and the structure recrystallizes during the process and, thus, allows the experimentally tested reductions to be applied without producing cold strain hardening, as can be seen in the stress–strain curves shown in Figure 13. As it can be observed, the stress–strain curves corresponding to each diameter sequentially processed by wiredrawing are practically overlapped with each other, which indicates that the material did not harden or modify its main mechanical characteristics even though it was cold deformed at low speed.

The alloy material’s yield strength (σ_d_) and plastic capacity were practically unchanged after each successive drawing pass. Definitively, this means that the material has been able to recrystallize during a process at room temperature and at low deformation speed. In this sense, a previous work consulted [45] demonstrated that plastic deformation processing in non-ferrous alloys, such as Mg–Si can give rise to intermetallic compounds of the carbide type along the grain boundaries, which can cause a concentration of residual stresses at the interface. According to the conclusions of the work by Jonda et al. [46], ZnAl15% alloys are composed of a solid solution (η) and a eutectic solid (η + α). Besides, a zinc-rich intermetallic precipitate is distributed along the grain boundaries which causes a slight surface hardening improving the mechanical properties of the alloy. This work demonstrated that the addition of Mg, Cu, and Fe induced the highest microhardness of the tested Zn and ZnAl15% samples, and it was found that Cd has a negative effect, decreasing the microhardness of Zn and the ZnAl alloy. In our case, a slight increase in the value of the maximum tensile stress has been observed as a greater degree of plastic deformation has been applied (Figure 13), which could be due to the presence of these intermetallic precipitates and Al content both subjected to plastic deformation.

## 4. Conclusions

As the main contributions in this work, an analytical model and a numerical simulation model (FEM) have been developed and refined, which may be useful for the redesign and optimization of multi-stage wiredrawing processes of the ZnAl15% alloy wire. For this purpose, the behavior model of the alloy when it is deformed by tensile stresses has also been defined, determining the strength coefficient (K) and the cold strain rate sensitivity coefficient (*m*) by means of which the effect that the deformation speed has on said behavior has been implemented, since zinc is a metal very sensitive to the deformation speed and, consequently, sensitive to variations in the strain rate during cold deformation.

It has been possible to verify, by comparison with the drawing stress values (σ_d_) obtained experimentally for the last five dies/stages of the wiredrawing sequence under study, that the model developed in the Deform 2D/3D v.12 FEM simulation software environment is very faithful to the experimental results, while the classic analytical model has given drawing stress (σ_d_) values that show a noteworthy divergence of up to ±10% with respect to those values recorded in real tests. Thus, the numerical model developed by Deform 2D/3D v.12 FEM software has mainly allowed determination of the values of drawing stress (σ_d_), drawing force (F_d_), and required power (P) for each of the 17 stages of the sequential process that is the subject of this study, with a high degree of confidence. This information could be very valuable for the design and development of manufacturing equipment. In addition, it is a model that has been refined and can be used for detailed analysis and to implement possible improvements in the sequential wiredrawing process of this very particular alloy.

Specifically, it has been determined that 2α = 10°, considering a shape factor Δ ≅ 4.00, corresponds, theoretically, to better geometry for the reduction zone to be used in the dies set of the multi-stage sequential wiredrawing process of the ZnAl15% alloy wire but, in the practice and by the recommendations of the die supplier, dies with 2α = 15° have been used in the experimental tests. This increase in the reduction angle (2α) supposes a larger contact surface between the die–wire in the reduction cone area, which prevents materials from being dragged into the die entry area and results in more even wear on said contact surface. In addition, establishing the maximum area reduction ratio (r) as being dependent on the yield limit of the alloy (σ_Y_) and considering the limit strain (ε) under this condition, it has been determined that r = 22.5% when it is intended to process the ZnAl15% alloy by wiredrawing. Thus, it has been corroborated that the industrial process designed by Victory Technology International Ltd. [36] is based on a conservative wiredrawing sequence, since the number of stages in which it is constituted implies a reduction ratio of between r = 8% and r = 15%, values that are very far from the maximum admissible area reduction in the alloy. This fact has been confirmed experimentally, observing that the mean value σ_d(mean)_ = 79.16 MPa of the drawing stresses achieved in the last five stages of the sequence taken as a reference is still far from the average value of the yield limit of the material at those same stages (σ_Y(mean)_ = 132.2 MPa). Consequently, this indicates that the industrial process could be improved in terms of reducing the number of stages/dies, saving technical costs but assuming a higher risk of wire breakage during the production process.

Furthermore, it has been confirmed that the coefficient of friction (µ) is a determinant in this type of process. In this sense, it has been determined with value of µ = 0.28 between the die made in tungsten carbide and the wire (ZnAl15%) when the wiredrawing is conducted at low speed, v = 0.003 m/s. Although this condition could be improved by a more efficient lubrication system and higher drawing speed, it has been proven that the processed material has had good dimensional and surface quality. Additionally, it has been confirmed that this alloy, with a high zinc content, reaches its recrystallization temperature during the process even at very low speed, since its fundamental mechanical properties modified (σ_Y_ and σ_YUTS_) have not been modified, showing absence of cold strain hardening. On the other hand, this work has validated the proposed models by limiting the production speed to a relatively low range (0.003 to 2.667 m/s), while industrial production speeds could be much higher and could represent a greater influence on the value of the drawing force required in each of the successive stages of the wiredrawing sequence. Thus, the results obtained in other works [27,44] that have been consulted and that were developed using simulation and also experimental methods invite the future development of the models proposed in this work for other higher speed ranges that confirm their industrial applicability.

This work has proposed a new methodology for the development of both analytical and numerical simulation models of the wiredrawing process of ZnAl15% alloy wires supported by simple experimental tests without the need for expensive industrial equipment. These models have proven useful in determining process limits and can, thus, be implemented for process analysis and optimization. Developed research expands knowledge and advances, facilitating the development of equipment, tools, and industrial processes in the multi-stage wiredrawing of ZnAl15% alloy wires. Furthermore, this work establishes a solid foundation that can help in the development of tools to optimize wiredrawing and advance the development of more efficient and sustainable processes, highlighting its ability to reduce process stages, decrease production costs, and energy consumption.

## Figures and Tables

**Table 1:** Chemical composition of ZnAl15% alloy according to UNE-EN ISO 14919:2024, in mass %, where total refers to the sum of impurities.

Si	Pb	Fe	Cd	Sn	Cu	Pb + Cd	Total	Al	Zn
0.12	0.005	0.05	0.005	0.001	0.01	0.006	0.17	14–16	Rest

**Table 2:** Numerical data from the tensile tests for adjustment by least squares to determine the strain rate sensitivity coefficient (*m*) of the 2.50 mm diameter ZnAl5% raw wire.

Speed, *v* [mm/min]	Yield Stress, σ_Y_ [MPa]	$\mathrm{Strain}\;\mathrm{Rate},\;\dot{\varepsilon }$ [s^−1^]	x log ($\dot{\varepsilon }$)	y log (σ_Y_)
6.25	144.76	0.001041667	−2.98	2.16
25	148.71	0.004166667	−2.38	2.17
50	147.94	0.008333333	−2.08	2.17
200	152.95	0.033333333	−1.48	2.18
		Ʃ	−11.33	8.69

**Table 3:** Industrial setup for the multi-stage wiredrawing process of the ZnAl15% wire proposed by the machine manufacturer Victory Technology International Ltd. (courtesy of Victory Technology International Ltd., Dongguan, China).

Stage/Pass nr.	Input Diam. d_o_ [mm]	Output Diam. d_f_ [mm]	Drawing Speed v [m/s]	Die Angle 2α [°]	Bearing Length L_c_ [mm]	Reduction Ratio r (%)
1	4.25	3.93	0.675	18	1.01	14.32
2	3.93	3.77	0.734	15	0.62	8.07
3	3.77	3.61	0.799	15	0.60	8.10
4	3.61	3.47	0.869	15	0.57	8.07
5	3.47	3.32	0.946	15	0.55	8.08
6	3.32	3.18	1.029	15	0.53	8.13
7	3.18	3.05	1.119	15	0.50	8.05
8	3.05	2.93	1.218	15	0.48	8.08
9	2.93	2.81	1.325	15	0.46	8.09
10	2.81	2.69	1.442	15	0.44	8.09
11	2.69	2.58	1.568	15	0.43	8.08
12	2.58	2.47	1.707	15	0.41	8.12
13	2.47	2.37	1.896	15	0.39	8.08
14	2.37	2.27	2.063	15	0.38	8.09
15	2.27	2.18	2.245	15	0.36	8.10
16	2.18	2.09	2.442	15	0.34	8.09
17	2.09	2.00	2.667	15	0.34	8.43

**Table 4:** Drawing stress (σ_d_), drawing force (F_d_), and power (P) in the industrial sequence of 17 stages, all at 0.003 m/s (courtesy of Victory Technology International Ltd., Dongguan, China).

			Process Model:				Analytical			FEM			Experimental		
Stage Nr.	d_o_ [mm]	d_f_ [mm]	v [m/s]	2α [°]	L_c_ [mm]	r (%)	σ_d_ [MPa]	F_d_ [N]	P [kW]	σ_d_ [MPa]	F_d_ [N]	P [kW]	σ_d_ [MPa]	F_d_ [N]	P [kW]
1	4.25	3.93	0.003	18	1.01	14.32	71.77	873	0.0026	75.70	920.5	0.0028	--	--	--
2	3.93	3.77	0.003	15	0.62	8.07	57.53	643	0.0019	76.50	854.5	0.0026	--	--	--
3	3.77	3.61	0.003	15	0.60	8.10	57.56	591	0.0018	72.30	742.5	0.0022	--	--	--
4	3.61	3.47	0.003	15	0.57	8.07	57.53	543	0.0016	72.90	688.2	0.0021	--	--	--
5	3.47	3.32	0.003	15	0.55	8.08	57.57	500	0.0015	72.70	631.0	0.0019	--	--	--
6	3.32	3.18	0.003	15	0.53	8.13	57.71	460	0.0014	69.80	556.3	0.0017	--	--	--
7	3.18	3.05	0.003	15	0.50	8.05	57.59	422	0.0013	71.80	526.3	0.0016	--	--	--
8	3.05	2.93	0.003	15	0.48	8.08	57.66	389	0.0012	69.80	470.5	0.0014	--	--	--
9	2.93	2.81	0.003	15	0.46	8.09	57.72	357	0.0011	71.00	439.5	0.0013	--	--	--
10	2.81	2.69	0.003	15	0.44	8.09	57.76	329	0.0010	69.70	396.6	0.0012	--	--	--
11	2.69	2.58	0.003	15	0.43	8.08	57.75	302	0.0009	69.00	360.9	0.0011	--	--	--
12	2.58	2.47	0.003	15	0.41	8.12	57.88	278	0.0008	68.30	328.5	0.0010	--	--	--
13	2.47	2.37	0.003	15	0.39	8.08	61.88	274	0.0008	68.00	300.6	0.0009	76.10	309	0.0009
14	2.37	2.27	0.003	15	0.38	8.09	57.89	235	0.0007	65.40	265.5	0.0008	71.50	290	0.0009
15	2.27	2.18	0.003	15	0.36	8.10	57.92	216	0.0006	65.10	242.8	0.0007	76.70	286	0.0009
16	2.18	2.09	0.003	15	0.34	8.09	57.92	199	0.0006	65.10	223.3	0.0007	83.80	255	0.0008
17	2.09	2.00	0.003	15	0.34	8.43	58.69	184	0.0006	67.10	210.7	0.0006	87.50	275	0.0008
								ΣP	0.0204		ΣP	0.0245			

**Table 5:** Drawing stress (σ_d_), drawing force (F_d_), and power (P) in the wiredrawing sequence, at a production speed of 2.667 m/s (Victory Technology International Ltd., Dongguan, China).

			Process Model:				Analytical			FEM		
Stage Nr.	d_o_ [mm]	d_f_ [mm]	v [m/s]	2α [°]	L_c_ [mm]	r (%)	σ_d_ [MPa]	F_d_ [N]	P [kW]	σ_d_ [MPa]	F_d_ [N]	P [kW]
1	4.25	3.93	0.689	18	1.01	14.32	76.80	934	0.643	79.10	961.9	0.663
2	3.93	3.77	0.750	15	0.62	8.07	61.61	688	0.516	73.40	819.9	0.614
3	3.77	3.61	0.816	15	0.60	8.10	61.74	634	0.517	74.70	767.2	0.625
4	3.61	3.47	0.888	15	0.57	8.07	61.77	583	0.517	73.30	692.0	0.614
5	3.47	3.32	0.966	15	0.55	8.08	61.89	537	0.518	71.40	619.8	0.598
6	3.32	3.18	1.051	15	0.53	8.13	62.10	495	0.520	69.50	553.9	0.582
7	3.18	3.05	1.143	15	0.50	8.05	62.04	455	0.519	65.80	482.3	0.551
8	3.05	2.93	1.243	15	0.48	8.08	62.18	419	0.521	65.60	442.1	0.549
9	2.93	2.81	1.353	15	0.46	8.09	62.32	386	0.521	65.50	405.4	0.548
10	2.81	2.69	1.472	15	0.44	8.09	62.42	355	0.522	64.10	364.7	0.536
11	2.69	2.58	1.601	15	0.43	8.08	62.48	327	0.523	63.80	333.7	0.534
12	2.58	2.47	1.744	15	0.41	8.12	62.87	302	0.526	63.80	306.2	0.534
13	2.47	2.37	1.896	15	0.39	8.00	62.51	276	0.523	75.30	332.8	0.631
14	2.37	2.27	2.063	15	0.38	8.09	62.83	255	0.526	72.70	295.2	0.608
15	2.27	2.18	2.245	15	0.36	8.10	62.94	235	0.527	75.10	280.1	0.628
16	2.18	2.09	2.242	15	0.34	8.09	63.01	216	0.527	74.30	254.8	0.622
17	2.09	2.00	2.667	15	0.34	8.43	63.91	201	0.535	70.60	221.7	0.591
								ΣP	9.009		ΣP	10.035

**Table 6:** Values of shape factor (Δ) of the drawing dies for die semi-angles (α) between 2° and 16° and area reductions (r) between 8.43% and 45%.

α [Rad]/[°]	Area Reduction (%)								
	8.43%	10%	14.31%	20%	25%	30%	35%	40%	45%
0.03491/2	1.59	1.33	0.90	0.63	0.49	0.39	0.32	0.27	0.23
0.06981/4	3.18	2.65	1.81	1.25	0.97	0.78	0.65	0.55	0.47
0.10472/6	4.77	3.99	2.72	1.88	1.46	1.18	0.98	0.82	0.70
0.13963/8	6.38	5.34	3.64	2.52	1.95	1.58	1.30	1.10	0.94
0.17453/10	8.01	6.69	4.57	3.16	2.45	1.98	1.64	1.38	1.18
0.20944/12	9.65	8.07	5.51	3.81	2.96	2.38	1.97	1.66	1.42
0.24435/14	11.32	9.47	6.46	4.47	3.47	2.80	2.32	1.95	1.67
0.27925/16	13.02	10.89	7.43	5.14	3.99	3.22	2.66	2.25	1.92

**Table 7:** Elongation from FEM simulation (e, FEM), associated with the area reduction (r) and the difference with theoretically calculated elongation (e).

r [%]	d_o_ [mm]	d_f_ [mm]	L_0_ [mm]	L_F,_ FEM [mm]	e, FEM [%]	e [%]	Difference
8%	1.974	1.894	20	22.249	11.25%	9.00%	2.62%
15%	2.054	1.894	20	24.311	21.55%	17.61%	3.94%
20%	2.117	1.894	20	26.018	30.09%	24.93%	5.16%
25%	2.187	1.894	20	27.848	39.24%	33.33%	5.91%
35%	2.358	1.894	20	33.262	66.31%	55.00%	11.31%
43%	2.500	1.894	20	wire breakage			

## Data Availability

All the original data are included in the article; further inquiries can be directed to the corresponding author.
